# Fertility of Cryptorchid Testis—An Unsolved Mistery

**DOI:** 10.3390/genes12121894

**Published:** 2021-11-26

**Authors:** Carmen Iulia Ciongradi, Ioan Sârbu, Codruța Olimpiada Iliescu Halițchi, Diana Benchia, Klara Sârbu

**Affiliations:** 1 2nd Department of Surgery—Pediatric Surgery and Orthopedics, “Grigore T. Popa” University of Medicine and Pharmacy, 700115 Iași, Romania; carmen.ciongradi@umfiasi.ro (C.I.C.); diana_benchia@email.umfiasi.ro (D.B.); 2 Department of Mother and Child Medicine-Pediatrics, “Grigore T. Popa” University of Medicine and Pharmacy, 700115 Iaşi, Romania; 3 Klara Sârbu MD Office, Târgu-Neamț, 615200 Neamț, Romania; klarasarbu@yahoo.com

**Keywords:** testis, undescended testis, fertility, cryptorchidism, orchidopexy, spermatogonia, hormonal, GnRH

## Abstract

Cryptorchidism (undescended testis) is one of the most common diagnoses in the pediatric urologist office. Even in the modern era, there still are a lot of debates regarding the optimal time for surgery related to the expected results in relation with the testicular function, including fertility. The review below intends to clarify issues regarding the impact of cryptorchidism on testicular histology and function, semen analysis, the relation between hormonal and surgical treatment, future fertility, and paternity rate.

## 1. Introduction

Cryptorchidism or undescended testis (UDT) is defined as unilateral or bilateral absence of the testes from the scrotum and it is the consequence of the testicular failure to descend into the scrotum prenatally. It occurs in 2.4–5% of newborns, is more common in preterm children (up to 30%), and decreased to 1–2% within three months when spontaneous descent is possible. The process is also less likely to occur after the age of six months [1,2]. Cryptorchidism is associated with high rates of male infertility, as nearly 10% of males with fertility issues have a history of UDT and orchidopexy, and 20–27% of male adults affected by azoospermia and 3–8% affected by oligo-terato-asthenospermia were previously diagnosed with testicular maldescent [3]. The incidence of infertility in unilateral cryptorchidism may be as high as 32% and goes up to almost double in bilateral UDT, even if surgically corrected. If left untreated, about 89% of patients with bilateral cryptorchidy will develop azoospermia [4]. Cryptorchidism plays an important role in male infertility, acting as a major risk factor with impact on testis anatomy and histology, impairing the reproductive function. The aim of this paper is to review the latest information about cryptorchidism causes, treatment, and implications in male fertility.

## 2. Factors Controlling Normal Descending of the Testis

Testicular maldescent is frequently and easily diagnosed, but understanding the mechanism of testicular descent continues to be one of the most challenging topics in pediatric urology. Some investigators consider that testicular descend is a biphasic process, controlled both by hormonal and mechanical factors. During early phases of gestation, the testis develops on the ventromedial surface of the mesonephros, and is anchored cranially by the suspensory ligament, and caudally by the genitoinguinal ligament or gubernaculum [5]. The descending process starts by the eighth gestational week with the transabdominal stage and lasts until 15 weeks of gestation. This stage involves contraction and thickening of the gubernaculum ligament and degeneration of the suspensory ligament under the influence of androgens and mediated by the testis itself, with the secretion of factors, such as insulin-like 3 (INSL3) and G protein-coupled receptor (LGR8 or RXFP2) [6]. Cell proliferation and an increase in extracellular matrix determine an enlargement of the gubernaculum that becomes bulky and gelatinous [7]. During fetal abdominal growth, this enlarged structure helps in anchoring the testis to the future inguinal region, by the traction exerted on the urogenital ridge through the gubernacular cord (its proximal connection to the testis) [8]. Under the effect of INSL3, there is an increase in gubernaculum volume which causes dilatation of the inguinal canal [9]. This stage is considered controversial by some authors, as it has been suggested that the testis retains a stable position while the abdomen grows cephalad.

Hadziselimovic et al. considers that histological studies of sagittal sections do not show a migration of the enlarged gubernaculum as sustained by Hutson in his schematic drawings [10]. On the contrary, during the development, the epididimys will enlarge and be able to hold the testis towards the future inguinal region, helping in the descending process of both testicle and gelatinous gubernaculum from the posterior abdominal wall, which is adjacent to the kidney, caudally to the internal inguinal ring. Then, the gubernaculum will create space in inguinal area by dilation of the canal, allowing further descent of the epididimo testicular unit [11].

At the end of the transabdominal phase, the testicular descent will pause until about week 25 of gestation, when the second phase, the inguinoscrotal one, starts, which will then finish around week 35. During this phase, the processus vaginalis (PV) seems to have a leading role. This structure develops within the gubernaculum, as a peritoneal diverticulum is stimulated by the abdominal pressure, a factor which seems to be important in the testicular descent at this time. The intraabdominal pressure stimulates PV formation through a weakness area in the inguinal triangle, which allows passage of the testis along the PV. This theory is supported by a higher rate of cryptorchidism in male infants with low abdominal pressure during fetal life, like those with abdominal wall defects [12,13].

Germ cells in different stages of development are found in the male testis, together with supporting Sertoli cells and Leydig cells in the interstitium. The primordial germ cells originate from the yolk sac and suffer a migration process towards gonadal ridge, where they become incorporated into the sex cords [14]. In the normal testis, at about 2–3 months after birth, a physiological gonadotrophin and testosterone surge occurs, as well as other currently unknown hormones. Under normal physiological circumstances, the fetal stem cells in the testis are stimulated by this hormone surge to develop into primary type A dark spermatogonia (Ad spermatogonia). At around 4 years of age, a second important developmental step in the prepubertal testis is reached; at this time, meiosis begins and primary spermatocytes appear, with a proliferation of germ cell numbers. Spermatogenesis can then commence at puberty [15].

Recent literature sustains the idea that altered gene expression program involving protein-coding genes and long noncoding RNAs (lncRNAs) will cause hypogonadotropic hypogonadism in boys with UDT and altered mini-puberty. An increased abundance of long noncoding RNAs participating in epigenetic processes, including *AIRN*, *FENDRR*, *XIST*, and *HOTAIR*, were noticed by Hadziselimovic et al. The observed increase in familiar cryptorchidism may reflect an epigenetic phenomenon. In isolated congenital UDT, they report an incidence of hypogonadotropic hypogonadism as high as 70% [16].

## 3. Cryptorchidism Impact on Testicular Function and on Semen Analysis

Cryptorchidism may have long-term impacts on testicular function, even after successful treatment. The number of Ad spermatogonia is reduced in UDT, indicating a failure in the maturation process of the gametic cells which directly correlates to future spermiograms.

Furthermore, 28% of adult men with bilateral operated UDT have azoospermia, compared to almost all with persistent bilateral cryptorchidism. Normal sperm concentration will be found in 71% of operated unilateral cryptorchidism and only in 48% of men with persistent UDT. In bilateral cryptorchidism, surgery performed after 10 months, but before 4 years of age, led to a normal sperm count in 76% (50–93%), compared to 26% (9–51%) in cases with orchidopexy done after 4 years of age. This impact of timing was not so obvious in unilateral cryptorchidism: 75% (68–81%) versus 71% (61–80%) if operated between 10 months and 6 years versus 9–12 years [17]. The fact that abnormalities in semen analysis are found in more than half of the patients with unilateral cryptorchidism suggests that, even if one testicle is situated in the scrotum, the disease is in fact bilateral and may lead to serious andrological problems.

In men with idiopathic oligozoospermia and in cryptorchid patients, the sperm DNA damage is significantly increased. The pathogenesis of sperm DNA damage in these patients may include the involvement of the seminal oxidative stress, suggested by the finding of increased reactive oxygen species (ROS) levels [18].

The exposure of the UDT to high temperature was incriminated as a factor that damages the gonads before the completion of sexual maturation, but recent data are consistent with the idea that infertility due to cryptorchidism caused by germ cell loss is a consequence of alterations in the P-element-induced wimpy testis (Piwi) pathway and transposon de-repression [19]. Germ cell death and genomic instability, due to uncontrolled transposon activity, could explain the decreased germ cell count identified in boys with cryptorchidism. Genome-wide RNA profiling analysis performed by Hadziselimovic et al. showed that genes important for transposon silencing were not expressed in the group of cryptorchid boys with high infertility risk, but only in the low infertility risk and control groups [18]. Processing bodies that harbor Piwi proteins are contained in spermatogonia. Piwi proteins and Piwi-interacting RNAs are specifically associated with silence transposable DNA elements. Azoospermia and infertility are the result of de-repression of transposable elements due to the loss-of-function mutations in the Piwi pathway [20]. Male-specific sterility and de-repression of LINE-1 (L1) retrotransposons is caused by the deletion of gametocyte-specific factor 1 (GTSF1), a protein involved in Piwi-mediated transcriptional repression. In the high-infertility-risk group, significantly lower RNA signals were found in seven members of the Tudor gene family and three members of the DEAD-box RNA helicase family. When compared to the high-infertility-risk samples, patients from the low-infertility-risk group showed stronger staining for GTSF1 and PIWIL4 proteins and weaker staining for the L1 transposon in the immune histochemical analysis. The data above suggest that cryptorchidism-associated infertility is a consequence of alterations in the Piwi pathway and by the impaired testosterone function during mini-puberty-inducing de-repression of transposon [21].

## 4. Cryptorchidism Impact on Testicular Histology

In descended testis, a prerequisite for normal spermiogenesis and fertility seems to be the disappearance of gonocytes (fetal stem cell pool) and appearance of Ad spermatogonia (adult stem cell pool). In normal male infants, maturation of the testis occurs in two steps: in the first phase an increasing in gonadotropin secretion between 2 and 4 months of age will stimulate Leydig cells in secreting testosterone and gonocytes will transform into Ad spermatogonia, providing a self-replicating pool of cells important in sustaining future spermatogenesis. In cryptorchid children, the testosterone secretion does not increase, resulting in an inadequate transition of gonocytes into Ad spermatogonia [22]. As this process is ineffective and delayed, the establishment of the adult stem cell pool is delayed and the persistence of the fetal stem cell pool is prolonged. Much more, until the beginning of the second year of life, the germ cell count continues to be high because the reduction in the number of germ cells per tubule does not take place, which histologically gives a ‘false’ impression of normal cryptorchid testis. After this age, the total number of germ cells will reduce below normal.

In 10–40 percent of the boys with UDT older than two years, a testicular biopsy at the time of surgery showed a lower germ cell count per tubule [23], and there are data that show a correlation between the sperm count after puberty and the number of Ad spermatogonia identified at biopsy [24]. If no Ad spermatogonia were present in one or both testes at the time of orchidopexy, the obtained sperm count would be inferior compared with cases in which Ad spermatogonia were found in both gonads. In those with no Ad spermatogonia in both gonads, despite successful orchiopexy, later sperm count identified that oligo/azoospermia, irrespective of cryptorchidism, was unilateral or bilateral. More than that, azoospermia identified in operated cryptorchid patient can be obstructive, caused by frequently associated anomalies of epididymis or due to unsuccessful orchidopexy [24].

The failure of so-called “minipuberty” (the surge in luteinizing hormone releasing hormone (LHRH), which triggers a release of LH and an increase in production of testosterone), is common in children with UDT around 2–3 months of age, as shown by Gendrel et al., who found a significantly lower level of LH in children aged 2–3 months with either unilateral or bilateral cryptorchidism [25]. This will cause a Leyding cell hypoplasia related to insufficient hormonal stimulation and under-stimulated Leyding cells will not be able to increase testosterone levels enough to determine germ cell maturation. 

In cryptorchidism, gonocytes may persist until about 1.5 years of age because the process of gonocytes transformation to Ad spermatogonia, which normally begins in the third trimester of pregnancy and is completed after the sixth month of life, does not occur. The consequence will be the absence of Ad spermatogonia and, later on, germ cells will lack in testicular biopsies. In cryptorchid testis, alterations may occur as they are not quiescent organs and germinal cells will be lost progressively. The risk of germ cells losing is higher for intra-abdominal testis, probably due to the effect of high temperature exposure. Hittleman estimated a two percent risk of cell depletion after one year of life for germ cell and a one percent risk per month for Leydig cell loss [26].

The cryptorchid testis recognize other histologic abnormalities: the older the child at the time of biopsy, the more disorganized testicular interstitial structure, with higher fibroblast and collagen content and a smaller number of Leydig cells. Changes in the mean diameter of seminiferous tubes, as well as in the number of germ cells and fibrosis degree, were found by Suskind et al., with higher fibrosis and smaller tubular diameter in older patients, as confirmed by Park’s studies [27,28]. In cryptorchid testis, lesions at the tubular level include a high degree of lamina propria fibrosis associated with a thickening of the basal membrane, which is arguably related to the inhibited maturation/differentiation of Sertoli, Leydig, and peritubular cells. In dysgenetic testes, basal membrane thickening increases with age, suggesting that age by itself may have a detrimental effect [29].

In light of claiming certain endocrinal disturbances to cause the failure of testis descent, cryptorchidism could be considered a bilateral disease even when a testicle is in the scrotum, because it was exposed to both. This theory is supported by the fact that more than half of the testicles found in the scrotum at birth in cases of unilateral cryptorchidism are malfunctioning. In unilateral cryptorchidism, there are histological changes, even if often less pronounced, in the descended testis, suggesting that the defect is not a localized, unilateral one. If, regarding the UDT, the problem of gonocyte transformation to Ad spermatogonia is recognized, a delay in the contralateral testis would also be observed, even if transformation was complete by 12 months of age in a majority of cases [30]. 

In a study conducted by Schindler et al. in 1987, which analyzes the scrotal testicle in cases of unilateral cryptorchidism, 30.1% from 495 testicular biopsies showed important germ cell loss, while the other counts were in the low–normal range, but showed a significantly lower mean when compared to the biopsies from the scrotal testis in patients with unilateral anorchia. Among the 18 boys with unilateral and 24 boys with bilateral UDT, control biopsies were performed later after orchidopexy. This showed no improvement regarding the count of germ cells, both in the originally cryptorchid or in scrotal testis; however, an improvement in the tubular diameter was noticed [31]. The concept that congenital dysgenesis affects not only the UDT, but also the scrotal one is also suggested by Nistal et al. who reports the limited benefits of orchidopexy alone [32]. The fact that azoospermia is reported to the control group 25 times more often in unilateral cryptorchid patients sustains the idea that the defect is, in fact, bilateral, even if only one gonad is in a clinically abnormal position [33]. 

## 5. Cryptorchidism Early Surgical Therapy and Its Implication on Fertility

The ideal age for surgery in cryptorchidism has been up for debate for a long time, over the last few decades decreasing progressively, from more than 10 years of age in 1950s to 1–2 years of age in 1990. Currently, the recommendation indicates that surgery should be performed at the age of six months [34], as, after that age, it is less probable that an UDT will descend [35]. Because histological examination of UDT at the age of twelve months has already revealed a progressive loss of germ cells and Leydig cells, any kind of treatment leading to a scrotal positioned testis should be finished by that time, or eighteen months of age at the latest [36]. In the long term, normal spermatogenesis and fertility could be impaired by a small temperature difference of 2–3 degrees between the scrotum and the abdomen. Because there are also data to suggest that, at 1–2 years of age, the number of germ cells per tubule starts to decline below normal, early orchidopexy could prevent further damage to testicular tissue.

Even if, for now, there are still debates regarding the causative factor of testicular impairment in cryptorchidism, and an inherently and developmentally defective testicle affected by the exposure to higher temperature, it is generally accepted that the decreased number of developed Ad Spermatogonia will lead to inadequate future stem cells for spermatogenesis with lower sperm counts and subsequent infertility. Beside oncological considerations, the decision to operate early assumes that the process is at least partially reversible and that placing the testis into its physiologic position in the scrotum will at least prevent further deterioration.

There are some facts which favour early orchidopexy: in the 1970s, a paper of Ludwig et al. showed a progressive decrease in fertility rate with the delaying of surgery, with a 90% rate of fertility for boys with orchidopexy in the first two years of life, a 50% rate in years 3–4, and a 30% rate in years 9–12 [37]. 

When comparing cryptorchid boys with surgery performed before 6 months with those operated between 6 and 24 months, Hadziselimovic et al. demonstrated a negative correlation between age at time of orchidopexy and germ cell counts. In all the patients operated before the age of 6 months, a normal count of germ cells per tubule was found, compared to the lower numbers in the group with surgery between 6 and 24 months. When long-term data were analyzed, 20 years after surgery, the authors observed no correlation between the number of germ cell at the time of surgery and the total sperm count on semen analysis, which raises some questions regarding the value of testicular histology at the time of surgery in fertility prediction. Statistically, there were no significant differences between the two groups in terms of semen analysis (<6 months: 136 × 106 sperm/ejaculate; >6 months: 96 × 106 sperm/ejaculate, *p* = 0.28). Nevertheless, it is important to underline that 31% of cryptorchid patients with surgery before 6 months of age had a sperm count less than 40 million. When analyzing the presence of Ad spermatogonia at the biopsy, irrespective of the age at the orchidopexy, the authors observed that almost all the patients who developed this more mature cell had a normal sperm count, but those without transformation in mature cells were with oligospermia or azoospermia. The results above emphasize the importance of the presence of Ad spermatogonia and maturation, which is likely the key to future fertility, as well as the fact that treatment should be able to offer the chance to the testicle of undergoing those maturation changes.

Based on current information, it seems that the presence of Ad spermatogonia is the key for preserving future fertility in patients with cryptorchid testis, as there are few studies that clearly correlate these histopathologic findings with the paternity rates. It seems rather that those testicles are not normal from the beginning and that early orchidopexy is just a preventive gesture in order to avoid further damage to an already impaired tissue.

## 6. Cryptorchidism Hormonal Therapy and Its Implication on Fertility

### 6.1. Early Hormonal Therapy

During early puberty, the gonadotropin-releasing hormone system is essential for epididymo-testicular descent and for gonocytes transformation into fetal spermatogonia, so, in time, there were many attempts to identify a hormonal therapy in cryptorchidism, using choriogonadotropin (hCG), gonadotropin-releasing hormone (GnRH), or luteinizing hormone–releasing hormone (LHRH), either as a nasal spray or by intramuscular administration. However, despite some reports, there are no clear data which prove the efficiency of hormonal treatment. 

There are some studies which show the efficiency of Buserelin both in inducing testicular descend, alone or with an increase in the number of germ cells, provoking further development of the epididymis [38]. In 1993, Bica and Hadziselimovic found that GnRH treatment stimulates further epididymis development and completion of epididymo-testicular descent, by inducing increased testosterone secretion. In the majority of patients with no response to hormonal treatment, a small and underdeveloped epididymis was identified. The authors consider that, when appreciating the response rate, one should consider the position of the epididymo-testicular unit before treatment [39]. A long-term follow-up open study published in 2017 showed that the result of successful hormonal treatment was stable at 4 years after, and that 65% of the testes still descended [40]. Another study demonstrated that after, administration of the buserelin nasal spray, followed by hCG (intramuscular injection), there was a significant rise in the number of spermatogonia per tubule [41].

The efficiency of hormonal therapy on fertility status is not yet recognized universally. Cortez et al. reported a better response in children with surgical orchidopexy than in those treated with hCG or GnRH, suggesting that hormonal treatment may suppress the germ cells, as they have found a higher number of spermatogonia in the surgical group [42]. There are also reports related to a better sperm count in patients receiving orchidopexy compared to those receiving hormonal treatment alone [43].

Hormonal therapy is reported to have a beneficial impact on normal descended, contralateral testis. In 2006, a study investigated patients with unilateral cryptorchidism randomized into two groups: those surgically treated and those treated with long-acting analog of LHRH and HCG. Biopsies obtained from contralateral descended testes were analyzed and compared with controls showing that the number of germ cells per tubule in contralateral testes of patients surgically treated was significantly lower than the number of germ cells in spontaneously descending testes (*p* < 0.0001). Regarding the histology of the contralateral testis, there were no adverse effects of hormonal therapy. On the contrary, the number of germ cells per tubule increased (*p* < 0.05) after seven weeks of treatment. A beneficial effect on the number of Ad spermatogonia per tubule and primary spermatocytes was also noticed, but those differences were not statistically significant [44].

European guidelines note that there is no consensus on hormonal treatment for testicular descent but suggest offering endocrine treatment for bilateral UDT to possibly improve further fertility potential [36]. 

### 6.2. Post Orchidopexy GnRh Therapy

As the main problem in cryptorchid testis seems to be the lack of gonocyte transformation in Ad spermatogonia as a consequence of impaired minipuberty, there were attempts to improve testicular histology and to increase fertility with hormonal treatment after surgical orchidopexy. Intra-nasal administration of buserelin (10 μg every other day for 6 months) in children with successful surgery showed a significant increase in the spermatozoa number per ejaculate, the percent of normal forms of spermatozoa, and sperm motility when compared to the control group with successful surgery alone [45].

Another study included thirty unilateral cryptorchid boys who were, on average, 3 years of age at the time of surgery and biopsy. The results of biopsy showed the impairment of testicular maturation and they were considered at high risk for infertility. Half of them were treated with 10 μg of buserelin nasal spray, administered on alternate days during 6 months, following surgery; the other half were the control group, who whose treatment was exclusively surgical. After puberty, at a mean follow up of 19 years of age, the semen analysis showed that the hormone-treated group had significantly higher concentrations of sperm (*p* = 0.000008) [46]. 

The use of hormone therapy in the management of cryptorchidism remains a considerable controversy, but a beneficial role of adjuvant GnRH analogues in improving fertility parameters seems to have a statistically significant evidence [47].

### 6.3. Adverse Effect of Hormone Therapy on Future Fertility

Several reports are showing that hormonal therapy may have adverse effects on testicular tissue. hCG administration in varying doses to male prepubertal Wistar rats determines adverse effects on both testosterone levels and germ cell haploid cell population [48]. It seems that local activation by Leydig cells and pro-inflammatory cytokines production by the resident macrophages acts as an explanation of hCG-induced testicular inflammation in rats. The authors speculated that cytokine-mediated inflammation of the testicle with secondary adverse effects on its function could be the result of high repeated doses of hCG used in the treatment of cryptorchid boys [49].

It is demonstrated that withdrawal of hCG (and/or androgen) increases germ cell apoptosis in the human testis [50]. Analyzing apoptotic DNA fragmentation in testicular biopsy specimens taken during orchidopexy in 25 adults with a history of cryptorchidism, Dunkel et al. showed that, in patients without hCG treatment, only few scattered apoptotic spermatogonia were identified by end-labelling of biopsies, whereas more extensive labelling of spermatogonia was seen in the 15 patients with a history of hCG therapy. The was a negative correlation between low-molecular-weight DNA fragmentation and testicular volume, and a positive one with the FSH levels in serum 20 years after biopsy. This may suggest that the normal testicular development could be disrupted by the hCG treatment, arguably through apoptosis [51]. 

## 7. Cryptorchidism and Its Implication on Infertility Management and Paternity

Cryptorchidism is associated with impairment of germ cell maturation and subsequent infertility in adulthood. Besides non-obstructive oligo or azoospermia, some studies indicate a high rate of obstructive patterns in patients treated for cryptorchidism. The association of seminal duct anomalies (from rete testis to vas deferens) with UDT are well known [52]. Congenital epididymal or vasal anomalies have a prevalence of 36–43% in patients undergoing orchidopexy [53,54], and biopsy of the testes with severe anomalies of ductal fusion shows the preservation of germ cells in 69% and diminished germ cells in 31% [55]. The prevalence and the severity of these anomalies appear to be strictly correlated to the localization level of UDT.

The other fact that can be involved in the etiology of obstructive azoospermia associated with UDT is related to surgical treatment, including the obstruction by iatrogenic vas deferens transection or excessive tension and aggressive manipulations of the spermatic cord [56]. It seems that about half of the operated patients (with normal spermatogenesis or mild hypospermatogenesis) have some form of obstruction that can be the primary or associated cause of azoospermia. 

Currently, no specific rational therapeutical approach is available for an infertile patient with a history of cryptorchidism; techniques of assisted fertilization could be considered and applied as symptomatic treatment. The spontaneous conception rate for infertile couples that recognize unilateral cryptorchidism as a causal factor is very low (about 1% per cycle). Advancements in sperm retrieval techniques permitted to extend the possibility of infertile couples to become engaged in the discussion of their potential for fertility.

Sperm with an intrinsically low fertilizing ability to produce viable embryos may now benefit from the technological advancement in micromanipulation techniques, such as intracytoplasmic sperm injection (ICSI), which may be utilized during in-vitro fertilization (IVF). The refinement and advancement of microsurgical sperm retrieval technique combined with ICSI, and their approval as accepted treatments for the azoospermic man, led to successful cycles, with excellent reported results on fertilization rates and pregnancy outcomes [57]. 

A study evaluating the conception rates in 48 infertile couples with unilateral UDT as a causal factor was performed by Mahmoud et al. It also evaluated 774 spontaneous cycles and 87 cycles of assisted reproduction. Similar success rates of 6.1% and 8.7% per cycle/attempt, respectively, were reported for intra-uterine insemination (IUI) and conventional in vitro fertilization (IVF). Pregnancy was obtained after ICSI in 46.7% of attempts, and sperm requirements for the latter treatment to be successful were lower than for IUI. Subfertility in men with unilateral cryptorchidism is severe, conventional IVF has little advantage, but ICSI is highly successful [58].

The most common cause of non-obstructive azoospermia (NOA) in men is represented by cryptorchidism. In cryptorchidism-associated azoospermia, the recovery of testicular spermatozoa (TESE) for ICSI is reported to be the only option. Moreover, 79 men surgically treated for cryptorchidism had other pathology. Thus, testicular failure was excluded and those with histologically proven NOA were included and treated (TESE with ICSI). In 41 patients (52%), testicular spermatozoa were recovered. In patients with a positive sperm recovery, the mean age at surgery was 10.6 years versus 15.5 years for those where no spermatozoa were obtained. In bilateral cryptorchidism, testicular spermatozoa were recovered in 21 of the 49 patients (43%), and 9 out of the 16 (56.2%) patients (*p* = 0.4) reported unilateral cryptorchidism. The sperm recovery rate for patients with a history of orchidopexy was ~50% in the population of men with non-obstructive azoospermia. Currently, there are no clinical parameters predicting successful sperm retrieval and pregnancy rates in this subpopulation of patients [59,60].

Another study included 38 cryptorchid patients who underwent 47 sperm extraction attempts to recover spermatozoa for ICSI. In 74% of attempts, spermatozoa were obtained, while 46% of these cases reported clinical pregnancy. When compared with noncryptorchid patients with non-obstructive azoospermia, the results were similar, if not better, in patients treated for UDT. Testicular volume (*p* < 0.05) and patient age at orchiopexy (*p* < 0.001) correlates well with spermatozoa recovery, but this one seems to be independent of serum FSH values. The data above suggest that testicular volume and age at the time of surgery are independent predictors of sperm retrieval for men with a history of cryptorchidism [60].

In a retrospective study comparing TESE results for 42 couples undergoing IVF treatment, Wiser et al. demonstrate that age at orchidopexy, above or below 10 years of age, was not a predictive factor for successful TESE. No differences in the sperm retrieval, fertilization, implantation, pregnancy, or live birth rates were identified between the two groups. It was found that sperm retrieval attempts in bilateral cryptorchidism, which are usually considered a testicular secretory dysfunction, yielded spermatozoa in almost 60% of patients with azoospermia and a history of cryptorchidism [61]. 

Future directions in the field of fertility improvement may also include techniques which already seem promising in animal models and can utilize in prepubertal oncological patients, such as spermatogonia stem cell transplantation. As already confirmed in rodent models with induced cryptorchidism translated to humans, this technique may be promising, at least for patients with severely damaged UDT and scarce spermatogonia [62,63]. 

Another issue related to fertility involves defining new markers of normal spermatogenesis dynamics. Recent studies found that proteins, such as the formin, disheveled-associated activator of morphogenesis 1 (DAAM1) regulating actin polymerization, and the prolyl endopeptidase (PREP) protease acting in microtubule-associated processes, are involved in cytoskeletal dynamics which are mandatory for normal testicular development, but also in pathological cell differentiation [64,65].

## 8. Conclusions

Cryptorchidism has a major impact on the fertility of affected male patients. Unilateral UDT is a bilateral disease, as histopathologic abnormalities can be found in contralateral scrotal testis. From the data above, it is fairly clear that, in cryptorchid testis, there is a defect in the transformation of the fetal gonocytes to Ad spermatogonia which may not be exclusively due to their ectopic location, but more to the failure of “mini-puberty”, so efforts should be made to preserve germ cell development, beside performing early orchidopexy, as hypogonadotropic hypogonadism seems to be the most common cause of cryptorchidism. Biopsy of the testis at surgery can provide data related to those testicles which may benefit from hormonal treatment, i.e., those lacking in Ad spermatogonia, as LHRH analogs induce replication and differentiation of germ cells which can then enhance the chance of future fertility. Future potential treatment strategies should focus on the possible prevention of abnormal gonocyte maturation, and long-term studies are still needed to correlate fertility and paternity rates with early orchidopexy. 

## Data Availability

Not applicable.
